# Progress in the Development of Flexible Devices Utilizing Protein Nanomaterials

**DOI:** 10.3390/nano15050367

**Published:** 2025-02-27

**Authors:** Chunhong Zhang, Chenxi Zhang, Yongchun Liu

**Affiliations:** 1 Xi’an Key Laboratory of Advanced Control and Intelligent Process, School of Automation, Xi’an University of Posts & Telecommunications, Xi’an 710121, China; 2 Key Laboratory of Applied Surface and Colloid Chemistry, Ministry of Education, School of Chemistry and Chemical Engineering, Shaanxi Normal University, Xi’an 710119, China

**Keywords:** protein, nanomaterials, flexible device, sensors

## Abstract

Flexible devices are soft, lightweight, and portable, making them suitable for large-area applications. These features significantly expand the scope of electronic devices and demonstrate their unique value in various fields, including smart wearable devices, medical and health monitoring, human–computer interaction, and brain–computer interfaces. Protein materials, due to their unique molecular structure, biological properties, sustainability, self-assembly ability, and good biocompatibility, can be applied in electronic devices to significantly enhance the sensitivity, stability, mechanical strength, energy density, and conductivity of the devices. Protein-based flexible devices have become an important research direction in the fields of bioelectronics and smart wearables, providing new material support for the development of more environmentally friendly and reliable flexible electronics. Currently, many proteins, such as silk fibroin, collagen, ferritin, and so on, have been used in biosensors, memristors, energy storage devices, and power generation devices. Therefore, in this paper, we provide an overview of related research in the field of protein-based flexible devices, including the concept and characteristics of protein-based flexible devices, fabrication materials, fabrication processes, characterization, and evaluation, and we point out the future development direction of protein-based flexible devices.

## 1. Introduction

Today, flexible electronics has received widespread attention in biomedicine, intelligent manufacturing, bioelectronics, and other fields [1,2,3,4]. Protein, the basic structural and functional unit in the life system [5], has become an important raw material for the preparation of new flexible electronic devices. Due to their rich structural and functional diversity, protein materials share excellent mechanical properties, adaptability, and low immunogenicity. By combining proteins with a variety of materials, not only can the sensitivity and conductivity of the device be improved, but signal transmission can also be promoted, and the stability, mechanical strength, and energy density of the device can be enhanced [6,7]. In recent years, with the rapid development of materials science, biochemistry, and nanotechnology, the internal structure of proteins has been modulated by designing the molecular structure, preparing heterostructures, and introducing dopants, which have significantly enhanced the physical and chemical properties of flexible devices.

Natural protein materials, such as silk fibroin (SF), collagen, and ferritin, exhibit unique physical and chemical properties that make them highly suitable for advanced applications. By compositing proteins with other materials, their inherent properties can be enhanced, significantly expanding their application potential. The resulting protein-based flexible devices demonstrate excellent electrical and mechanical properties, making them ideal for the development of biosensors and memory devices, particularly due to their inherent pressure sensitivity and insulating characteristics [8]. To further improve device performance, proteins can interact with carbon materials through hydrogen or covalent bonds via functional groups, which enhances stability, electrical conductivity, and mechanical strength [9,10,11]. Additionally, proteins can be combined with metal nanoparticles through physical embedding or electrostatic adsorption [12], enabling the fabrication of stable and flexible sensors and memory devices. Moreover, advanced techniques such as in situ polymerization, layer-by-layer deposition, and functional ink printing allow proteins to be complexed with polymers, facilitating the development of high-performance sensors, non-volatile memories, and flexible capacitors. These diverse preparation strategies provide a robust technical foundation for the multifunctionalization and efficient application of protein-based flexible devices. This versatility underscores the significant potential of protein materials in next-generation flexible electronics.

Protein materials, with their unique biocompatibility, biodegradability, and mechanical flexibility [13], exhibit significant potential in sensing, energy storage, and bioelectronic memory applications. In sensing, protein-based devices utilize piezoelectric, capacitive, and piezoresistive effects to convert mechanical signals into electrical signals, enabling the precise detection of pressure and physiological signals [14,15]. Additionally, protein-based humidity sensors operate by adsorbing water molecules, triggering conductivity or capacitance changes, and are self-powered, making them ideal for medical, agricultural, and environmental monitoring. For energy storage, proteins and their composites enhance the specific capacitance, energy density, and stability of supercapacitors and piezoelectric devices [16]. These improvements address the growing energy storage demands of wearable electronics and low-power devices, offering sustainable solutions for next-generation energy systems. In bioelectronic memory, proteins serve as switching layers or storage media in memristors and transistors, achieving high switching ratios and stable non-volatile storage behaviors through redox reactions or conductive channel formation [17]. This capability advances the development of brain-like computing and biocomputers, paving the way for innovative computing architectures. These versatile applications highlight the transformative potential of protein-based flexible devices in next-generation technologies.

**Figure 1:**
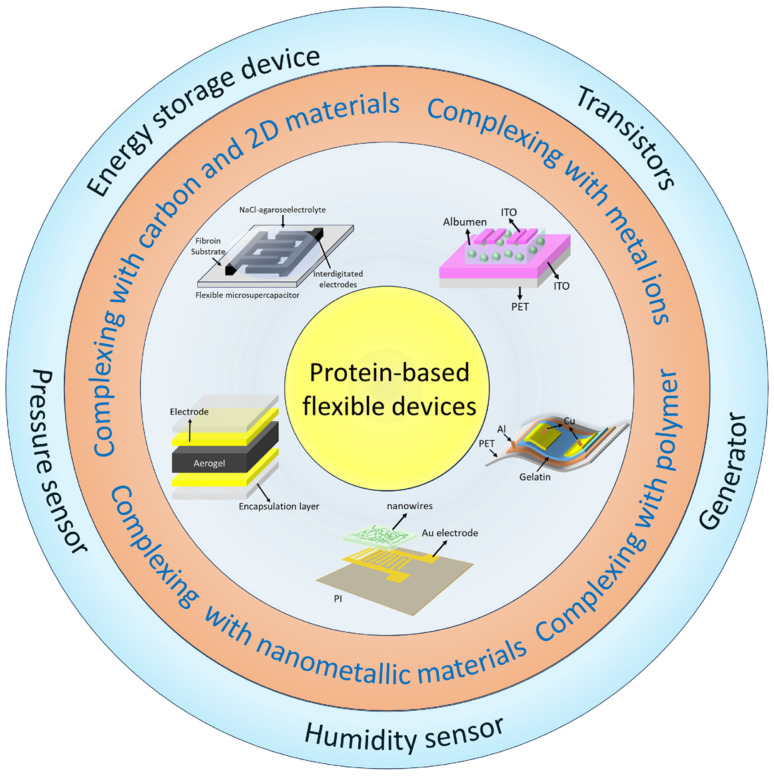
Applications of protein-based flexible devices.

In this paper, the preparation process, fabrication materials, basic principles of protein-based flexible devices, and application areas are reviewed (Figure 1). In terms of preparation strategies, the physicochemical properties of natural proteins and their advantages as raw materials for flexible electronic devices are explored, and the composite methods of proteins with carbon materials, two-dimensional (2D) materials, metal ions, metal nanomaterials, and polymers are investigated. In the application areas, protein-based flexible devices show a wide range of potential in sensing, energy storage, bioelectronic memory, and power generation devices, such as the use of piezoelectric, capacitive, and piezoresistive effects to realize precise sensing; and the use of piezoelectric, friction electric, and hydrovoltaic effects to realize self-power supply and green energy conversion. This paper provides important theoretical support and technical insights for the research and development of protein-based flexible devices.

The paper is structured as follows: Section I mainly summarizes the research progress of protein-based flexible electronic devices and their application potential. Section II describes the preparation strategies for flexible devices. The properties of the protein materials themselves and their composites with other materials are explored. Section III describes the applications of protein-based flexible devices. The applications of protein-based flexible devices and their properties in the fields of sensors, energy storage devices, and electronic bio-memory devices are listed. Section IV summarizes the entire paper. It mainly summarizes the application of protein materials in flexible electronic devices and their advantages. It also provides an outlook on the future development of protein-based flexible devices.

## 2. Preparation Strategies for Flexible Devices

Compared to synthetic materials, natural polymeric materials, especially protein-based biomaterials, are the most attractive alternatives due to their inherent properties, which can better address the challenges faced by synthetic materials. In the research and development of protein flexible devices, protein materials such as SF, ferritin, and collagen have attracted much attention due to their unique physical and chemical properties (Table 1). By compounding with carbon materials, 2D materials, metallic materials, and polymers, protein materials together constitute the multifunctionality of protein flexible devices, which makes them have a wide range of applications in many fields, such as smart sensing, bioenergy storage, information storage, and so on.

### 2.1. Electrical Properties of Proteins

Proteins not only perform important biological functions in living organisms but also show great potential in the field of electronic devices due to their unique electrical properties. In 1977, Hill et al. observed the direct electrochemical behavior of proteins for the first time [24]. Exploring the direct electron transfer process of redox proteins at the electrode interface and combining them with other materials to assist in enhancing the performance of the device have far-reaching theoretical and practical significance for the development of new bioelectronic devices and the exploration of physiological activities and their operation laws in living organisms [25]. For example, the cytochromes in bacterial nanowires are able to perform electron transfer reactions according to their specific biological functions and have been observed to exhibit electron tunneling within a few millimeters [26]. Chen et al. demonstrated in detail the direct electron transfer behavior of hemoglobin on various nanomaterial-modified electrodes [27,28,29]. Xie et al. [30] successfully prepared a nanodiamond (ND) for the construction of a Nafion/Mb/ND/CILE sensor and used this myoglobin sensor for the detection of three to-be-measured substances: trichloroacetic acid, sodium nitrite, and hydrogen peroxide.

#### 2.1.1. Pressure-Sensitive Properties of Proteins

On this basis, more and more scholars have begun to focus on the pressure-sensitive properties of proteins under mechanical pressure to further explore the potential of proteins in electron transfer efficiency and functionalization and to promote the research and development of protein-based high-performance pressure-sensitive bioelectronic devices.

**Figure 2:**
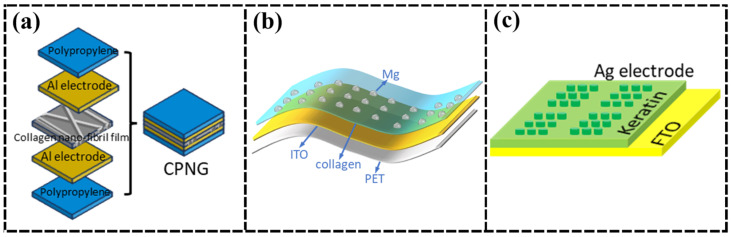
Pressure-sensitive properties of proteins themselves. (**a**) A cost-effective self-powered, eco-friendly, and bio-compatible piezoelectric biopolymer [19]. (**b**) A collagen-based, flexible, and transient memristive device that is water-soluble [21]. (**c**) An Ag/Keratin/FTO ReRAM device with physically transient characteristics [22].

Silva et al. [18] studied the physicochemical and piezoelectric properties of collagen-chitosan films and concluded that the piezoelectric strain constant of collagen was 0.096 pC/N, and the piezoelectric property was improved to 0.212 pC/N after the addition of 15% (mass fraction) of chitosan, which is about 1/10 of the piezoelectric constant of quartz crystal. They also investigated a pressure sensor prepared on the surface of a collagen membrane for biological applications and looked ahead to its potential use in cardiovascular prostheses, support for cellular growth, and in systems for controlled drug delivery. On this foundation, Vivekananthan et al. [19] prepared a self-powered piezoelectric biopolymer humidity sensor (SP-PB-HS) using collagen-coated cotton fabric as a piezoelectric material (Figure 2a), demonstrating that piezoelectric collagen-nanofiber biopolymers coated on cotton fabrics have dual functions as energy harvesters and sensors. However, if the collagen nanofibers are not uniformly coated, they may lead to poor alignment of the electric dipoles, which reduces the piezoelectric properties, and the device may be polarized at higher electric fields.

Although protein materials have natural abundance, a wide range of chemical compositions, and excellent mechanical flexibility, they have weaker mechanical strength and lower elongation than most synthetic polymers. As a result, devices prepared with protein materials alone have limitations in terms of mechanical properties and are unable to fully utilize the potential advantages. Thanks to the synergistic effect between different materials, the unique advantages of various materials can be fully utilized by constructing a composite system to significantly enhance the overall performance of the device.

#### 2.1.2. Insulating Properties of Proteins

Proteins, as inert organic materials, are good bioinsulators. Studies have shown that proteins are able to form uniform and dense films and exhibit good electrical insulation properties in their natural state [31]. This characteristic gives proteins significant application potential in the field of electronic devices. For example, a protein film used as a dielectric layer in electronic devices can prevent current leakage and enhance the stability and reliability of the device. In wearable electronic devices, proteins can be used as an insulating layer to prevent electrodes from directly contacting human tissues, reducing stimulation or damage to human tissues and enhancing the safety and durability of the device. In biosensors, the protein insulating layer can effectively isolate the electrical signal, prevent interference, and improve the accuracy and stability of the signal. With the rise of bioelectronics and sustainable technology, researchers have tried to apply different types of protein materials to electronic devices. These protein-based films not only exhibit excellent insulating properties but also address the shortcomings of traditional materials in terms of flexibility, transparency, and biodegradation by modulating the material properties, which provides new ideas for the innovative design of future electronic devices, and also promotes the research and development of environmentally friendly functional materials.

Zeng et al. [20] extracted collagen from pig skin as an intermediate insulating layer and constructed bio-memory devices with two structures, Ag/Bio membrane/ITO, and Ag/Bio membrane/Ti. With ITO as the bottom electrode, the high-resistance state (HRS)/low-resistance state (LRS) resistance ratio is 10, while with Ti as the bottom electrode, the HRS/LRS resistance ratio can be increased to 100. However, both ITO and Ti are rigid materials. In order to solve this problem, Hosseini et al. [21] used a solution-assisted process to prepare a flexible Mg/collagen layer/ITO-structured transparent bio-memory resistors (Figure 2b). The flexible collagen-based bioresistors fulfill the basic requirements for reproducible nonvolatile memory, including data retention, durability, and mechanical flexibility under tensile and compressive stresses. Moreover, magnesium and collagen are soluble in water and can be completely dissolved in contact with water, making the device potentially promising for applications in storing and analyzing top-secret defense information. In contrast, Lin et al. [22] employed a keratin piezoelectric film as a solid electrolyte layer to prepare a memristor that exhibited excellent electrical performance, high transmittance, and good physical transient characteristics (Figure 2c). This keratin-based nonvolatile memory exhibits stable switching performance, centrally distributed switching voltages (±1.5 V), a switching ratio of more than 10^3^, and a retention time of 10^4^ s. In addition, the keratin film can dissolve in deionized water within 30 min, presenting potential biodegradability and physical transient characteristics of the memory device.

In addition to memristors, Wang et al. [23] prepared flexible organic thin-film transistors (OTFTs) on poly(ethylene terephthalate) (PET) substrates using SF as an insulating layer. Experiments have shown that the flexible OTFTs prepared using SF can achieve mobility as high as 23.2 cm^2^/V·s, an operating voltage as low as −3 V, and excellent stability. The structural properties of SF effectively improve the crystal quality of the organic semiconductor layer and reduce the interfacial trap density, which significantly enhances the performance of the devices.

Protein materials as insulating layers have unique flexibility, excellent light transmittance, and biodegradability, which can effectively prevent current leakage and enhance the reliability and safety of devices. However, the preparation and processing of protein materials are more complex than those of traditional inorganic materials, and the relative dielectric constant of protein materials is generally lower than 5, limiting their performance in high-frequency or high-electric field applications. In addition, the inherent insulating nature of proteins prevents them from being used as functional electronic materials alone, and they must be composited with other conductive materials (e.g., conductive polymers, carbon-based materials, or metal nanowires) to build heterostructures or hybrid systems to achieve effective charge transport and device functionalization.

### 2.2. Protein Complexes with Other Materials

Protein composite technology with other materials opens up new directions for innovative applications in electronics and biomedicine. By combining proteins with a variety of materials such as carbon materials, 2D materials, metal ions, nanometals, and polymers (Table 2), the mechanical properties, electrical conductivity, biocompatibility, and degradability of composites can be effectively enhanced, thus promoting the development of high-performance sensors, energy memory devices, smart electronic devices, and biomedical devices.

#### 2.2.1. Protein Complexes with Carbon and 2D Materials

Composite applications of proteins with carbon and 2D materials are of great research value. These materials combine the biocompatibility and degradability of proteins with the excellent electrical properties of carbon and 2D materials, providing a broad space for the development of high-performance sensors, energy memory devices, and smart electronic devices. Chemical groups in protein molecules form non-covalent bonds with materials such as carbon nanotubes (CNTs) and graphene to form a tight composite structure [51,52], which can significantly improve the mechanical strength, thermal stability, and electrical conductivity of the materials, and provide a new way of thinking for the design of green and sustainable wearable devices and biomedical devices.

Silk is solidified from the silk fluid secreted by silkworms and contains SF and silk sericin proteins, which are widely used in textiles, biomedicine, and drug delivery due to their excellent mechanical properties, biocompatibility, and controlled biodegradation [13]. Recycled SF-based materials have unique molecular structures and functional groups (e.g., amino, hydroxyl, carboxyl, etc.), which can be chemically altered by changing certain side groups or introducing other functional groups (e.g., sulfonic acid, phosphoric acid, epoxy, etc.) to be composited with CNTs [53], graphene, and silver nanowires (Ag NWs) to improve the mechanical properties, thermal stability, and functionality while preserving the structure of the SF main chain. These composites can be applied in the fields of bioengineering, tissue engineering, smart materials, and other fields. The natural affinity of ferritin for CNTs enhances the mechanical properties of polymers and electron transfer, with CNT–biomolecule interactions altering protein conformations and electrocatalytic functions [51]. For instance, functionalized single-walled CNTs (f-SWNTs) mixed with ferritin retain redox properties, enabling electron transfer within the ferritin shell, making f-SWNT/ferritin composites promising as potential biosensors [54]. Additionally, collagen, a naturally occurring fibrous protein with good flexibility, is capable of forming hydrogen or covalent bonds with carbon or 2D materials through chemical reactions. For example, collagen molecules can be covalently grafted in situ onto the surface of CNTs through the formation of amide bonds, thereby significantly improving the bending and fracture strength of CNTs [55]. The high carbon content in collagen can form a tight composite structure with the carbon material, further enhancing the overall performance of the composite.

**Figure 3:**
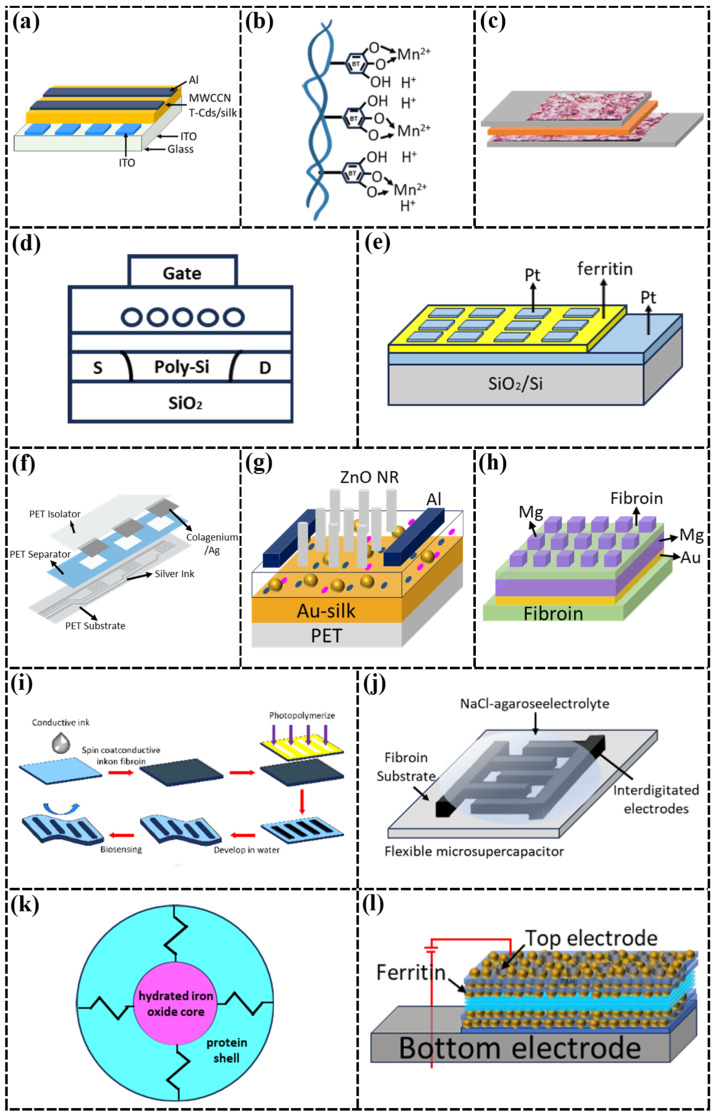
Protein complexes with other materials. (**a**) A carbon nanotube-CdS nanostructures/silk nanocomposite device [32]. (**b**) A Mn/N-C-x composite based on the inexpensive and abundant collagen fibers extracted from the offcuts of the leather waste [33]. (**c**) A high-performance energy storage system is constructed using extracted fish collagen [34]. (**d**) An LTPS-TFT flash memory with a ferritin core floating gate was fabricated using the bio-nano process [36]. (**e**) A biocompatible memristor device based on natural ferritin has been fabricated [37]. (**f**) Collagen/Ag NW composites for resistive sensor applications [38]. (**g**) ZnO photodetectors on Au-silk protein films for flexible optoelectronic devices [41]. (**h**) Physically transient resistive switching memory anodevices based on silk proteins [44]. (**i**) Micropatterned flexible devices using silk proteins [46]. (**j**) A green and sustainable flexible microsupercapacitor based on a conducting polymer [47]. (**k**) A wearable flexible strain sensor. (**l**) Layer-by-Layer assembled multilayers based on protein nanoparticles [48].

The advent of CNTs has revolutionized the development of nanomaterials [56], which have a wide range of applications in many research fields. CNTs have superior mechanical, electrical and thermal properties, such as high tensile strength, lightweight, high specific surface area, excellent electrical properties, and high thermal conductivity, which make them ideal materials for building functionalized fibers and smart flexible fabrics [57]. S. Bera et al. [32] combined nanostructured materials with SF substrates, embedding CNT-CdS nanostructures in a silk protein matrix to prepare floating gate memory devices. CNTs have excellent mechanical strength and chemical stability (Figure 3a), which can significantly enhance the overall strength of the composite material, and do not displace the metal electrodes during the bending process; they also have a high electrical conductivity and a large specific surface area, which are favorable for the efficient transmission of electrons, providing an avenue for flexible, transparent, and printable electronics.

In exploring 2D materials for biomedical applications, research and application of 2D layered materials have attracted much attention with the discovery of graphene. Among these, semiconducting transition metal dichalcogenides (TMDCs) are highly esteemed for their excellent electrical and optoelectronic properties. Tungsten disulfide is a type of TMDC that offers the advantages of biocompatibility, a tunable bandgap, and excellent optoelectronic properties. These properties make it a potential candidate material for chemical sensing, biosensing, and tumor therapy [58]. Lei et al. [33] extracted collagen fibers from collagen and successfully prepared manganese-doped nitrogen-containing carbon materials, applying them in supercapacitors (Figure 3b). The manganese-doped nitrogen-containing carbon materials are ideal candidates for making supercapacitor electrodes because of their high specific capacitance (272.62 F g^−1^), long service life, and good thermal stability under extreme conditions. Selvam et al. [34] prepared supercapacitor (SC) electrodes with a surface capacitance of 348 mF cm^−2^ using a collagen matrix extracted from blue tip cod skin doped with polypyrrole (PPy) and tungsten disulphide nanoparticles to provide the desired electrical performance (Figure 3c), which promotes the development of biocompatible and highly stable energy storage devices and provides a high-performance biomedical devices design with a new avenue for the design of high-performance biomedical devices.

As biological macromolecules, proteins have abundant functional groups on their surfaces that can form chemical bonds (e.g., π–π stacking, hydrogen bonding) with carbon or 2D materials to optimize interfacial contacts and reduce interfacial resistance for charge transfer. Therefore, compositing with carbon and 2D materials can improve the electrical and optical properties of the devices, while improving the catalytic activity of proteins through functionalized surfaces can lead to more efficient electron transfer. However, the emission and metabolism of carbon and 2D materials in the human body are still unclear and may trigger biotoxic reactions.

#### 2.2.2. Protein Complexed with Metal Ions

Research on ferritin as a core material has made remarkable progress in the fields of bioelectronics and nanotechnology in recent years. Some proteins are inherently capable of complexing with metal ions and thus perform important functions. The unique properties of ferritin provide new technological routes for the development of electronic devices such as bioinformation storage, memristors, and flash memory. Ferritin subunits are capable of self-assembling into nanocages, and the unique nanoscale internal cavities can efficiently load and transport a variety of substances such as transition metals, fluorescent molecules, and nanoparticles [59]. As a natural protein cage nanoparticle with enzyme-mimicking activity, ferritin can be easily coupled with biomolecules to construct nanobiosensors [60], and ferritin can be easily modified or altered chemically and biologically [61], and has good biocompatibility and targeting, among other features, which make it a non-viral natural nanomaterial with great potential for biomedical applications.

Precious metal ions have been widely used in organic synthesis, environmental protection, energy, and other fields due to their excellent catalytic activity, electrochemical properties and stability. Elmas et al. [35] prepared ferritin-based bio-memory substrates using photo-sensitive electron-transferring microemulsion copolymerization using the Amino Acid (monomer) Decorated and Light Underpinning Conjugation Approach method, which can undergo electron transfer through reduction and oxidation processes. The protein substrates contain metal ions or metal ion pairs such as Ag and Cu, i.e., copolymer shells of Ag, Cu, and photosensitive cross-linked proteins. Depending on these metal ions or metal ion pairs, the redox properties of the biomimetic carriers can distinguish between electrochemical “write” and “erase” states. The polymorphic memory devices constructed in this way have high stability and durability and represent a promising future for bioelectronic information technology. However, the complex preparation process and the stringent requirements for material selection and the synthesis process limit its mass production and practical application.

Iron ion is a metal cation, the most stable ion of iron, with strong oxidizing property, which is ideal for use as an active substance and plays a significant role in chemical catalysis, the biological field, and various disease detection and treatment [62]. Ichikawa et al. [36] proposed a “bio-nano-process” to prepare high-density homogeneous nanodots using ferritin without a vacuum system, providing technical support for the development of flash memory for system panels (Figure 3d). Ferritin adsorbs iron ions to form inorganic nanoparticles with a diameter of 7 nm, which are then adsorbed onto silicon thin films to prepare low-temperature polycrystalline silicon (poly-Si) thin-film transistors. In contrast, memristor devices are expected to become mainstream devices for future chips due to their advantages of high integration density, fast operation speed, low power consumption, integrated storage and computation, and excellent synaptic and neuronal plasticity. Zhang et al. [37] fabricated biocompatible memristor devices with a Pt/ferritin/Pt interlayer structure on a SiO_2_/Si substrate (Figure 3e). The release and adsorption of ferric ions control the switching between LRS and HRS for encoding “0” and “1” under the effect of an applied electric field, offering advantages such as a simple structure, low power consumption, fast operation speed, and suitability for three-dimensional ultra-high-density integration, with potential applications in information storage, logic circuits, and neuromorphic computing.

Complexation of proteins with metal ions provides the devices with excellent biocompatibility and tunable electron transfer properties, which endows the devices with polymorphic memory and good regulation. However, the conductive behavior of the devices is affected by the ion distribution within the protein, so the composite of protein and metal ions may lead to unstable conductive paths. It also faces problems such as complex processes and strict material selection requirements, which make it difficult to be mass produced and applied.

#### 2.2.3. Protein Composite with Nanometallic Materials

Proteins can significantly enhance their electrical, mechanical, and chemical properties by compositing with various nanomaterials. By compositing with various conductive materials, such as metal nanowires, nanoparticles, and metal polymers, proteins can form interfacial phases with a certain thickness at the interface through interfacial bonding, such as hydrogen bonding, covalent bonding, etc., which has an important impact on the full play of the properties of each component and the final performance and function of the composite material [63,64].

SF molecules have a considerable number of amino acid residues, such as threonine, aspartic acid, glutamic acid, lysine, tyrosine, etc., which can be modified and crosslinked by conventional chemical methods [65], laying the foundation for binding to metal nanoparticles. The metal nanoparticles have a large specific surface area [66], high surface activity, and exhibit properties such as the small size effect, the surface effect, and the quantum size effect, which can provide more reaction sites when composited with SF; at the same time, they have high electron density, dielectric properties, and catalytic effects [67], which can bind to SF at the molecular level without affecting its biological activity and thus improve the performance of the composites. SF at the molecular level without affecting its biological activity, thus improving the performance of the composites. Collagen is a green and renewable organic natural polymer material with active groups such as -COOH, -NH_2_, -OH, and -SH distributed on the molecular chain, which is easy to modify and has good biocompatibility, biodegradability, and low antigenicity, and has been widely used in clinical medicine, food processing, and cosmetics. However, protein membranes based on collagen still suffer from common problems such as insufficient mechanical strength, low barrier properties and weak electrical conductivity in applications. Introducing inorganic nanomaterials with unique functional properties into them can develop new collagen-based nanocomposites that combine the excellent properties of both [68].

In addition to the excellent electrical conductivity of silver, silver nanowires also have excellent light transmittance and flex resistance due to the nanoscale size effect [69]. Andonegi et al. [38] prepared collagen/AgNW nanocomposites with a maximum electrical conductivity of 0.0515 S cm^−1^ and presented a proof-of-concept for the applicability of collagen/AgNW nanocomposites as resistive sensors (Figure 3f), demonstrating the potential of sustainable and multifunctional antimicrobial composites based on the value of bio-based resources. In contrast, the SF main chain structure is less susceptible to damage from chemical reactions and contains a richer variety of reactive moieties, showing stronger potential for functionalization. Therefore, Chen et al. [39] further combined SF membranes with Ag NWs to prepare flexible capacitive sensors. The SF film exhibited excellent transparency, mechanical properties, and insulation, with tensile strength and elongation at break reaching 82.47 MPa and 11.2%, respectively. The device can work stably under normal environmental conditions and can be completely decomposed in one milliliter of deionized water, which has the advantages of low cost, environmental protection, and information security, and lays the foundation for its application in the field of flexible electronics.

Compared with silver, gold has stronger electrical conductivity, and the combination with protein materials can significantly improve the performance of devices. In addition, the rich polar functional groups of SF and gold nanoparticles adsorb each other through electrostatic interaction [70], which can form structurally stable nanocomposites. Therefore, Lee et al. [40] developed a highly tunable and fully biocompatible nano-plasmonic bio/chemical sensor based on natural SF and gold nanostructures by utilizing a metal–insulator–metal absorber structure, with strong interactions between amino acids in SF and gold, which significantly improves inter-materials adhesion and device stability, with spectroscopic sensitivity up to 1200 nm/RI. It provides an innovative platform for real-time, sensitive in vivo monitoring and biomedical research.

Also combining SF with gold nanoparticles, Gogurla et al. [41] developed a flexible and transparent memristor by using spin-coating to prepare SF-gold nanoparticle films (Figure 3g). This process is simpler, less costly, and suitable for large-scale fabrication. On the basis of memristors, Gogurla et al. [42] also prepared metal-semiconductor-metal (MSM) transverse photodetectors, where the presence of gold nanoparticles increased the light-dark current ratio from 5.5 × 10^3^ in ZnO devices to 3.5 × 10^5^, with a peak detection rate at 350 nm of 5.0 × 10^11^ Jones, which is 2.6 times higher than ZnO devices and has good mechanical stability under compression and stretching. Based on lightweight considerations, Wang et al. [43] used SF film as the switching layer to prepare the ultra-lightweight memory device, which is 320 times lighter than the traditional silicon substrate device, 20 times lighter than the office paper, and can be supported by only one hair. In order to meet the requirement of solubility, Wang et al. [44] also prepared transient memristors by using an Au/Mg/Silk/Filament/Mg structure, which can be completely dissolved in deionized water or a phosphate-buffered salt solution within 2 h (Figure 3h).

The synergy between the biofunctional diversity of proteins and the physicochemical tunability of nanometallic materials allows the composite of proteins with different nanometallic materials to tune the electrical, optical, and mechanical properties of the devices as needed, conferring flexibility to the devices. However, the preparation of nanostructures requires highly precise fabrication techniques, and any defects in fabrication can affect the performance of the sensors. In addition, nanometallic materials are prone to react when in contact with proteins, which may lead to interfacial defects, changes in contact resistance, or metal dissolution, which, in turn, affects the overall device stability.

#### 2.2.4. Protein–Polymer Complexes

Research on composites of proteins and polymers has opened up new technological and application pathways in the field of flexible electronics and bioelectronics. Composites combine the biological properties of proteins with the mechanical and electrical properties of polymers to achieve excellent functional properties and application performance through innovative design and processing methods. From multilayer composite membranes of ferritin and functional polymers to bio-storage devices based on protein carriers, these composites show high flexibility and stability in the fields of flexible sensors, non-volatile device storage, and biocompatible supercapacitors, which provide brand new solutions for the next-generation of flexible bioelectronics and degradable electronics. The related technologies and research progress are described in detail below.

PPy is an electrically conductive and highly stable polymer used in various types of sensors, supercapacitors, nanowire actuators, and more [71,72]. The study by Ke et al. [45] made innovations in cross-scale detection. Using cowhide as a flexible and stretchable substrate and PPy in situ growth treatment, a skin-friendly sensory skin was prepared with collagen composition and hierarchical structure features similar to human skin. The layered structure of bovine skin fibers can effectively transmit external forces along the direction of the fibers while generating cross-scale deformation to adapt to the strain caused by external forces, thus obtaining the dual ability to sense weak physiological signals and large-scale body movements under high strain.

Poly(3,4-ethylenedioxythiophene)/poly(styrenesulfonate) (PEDOT:PSS) solution has good electrical conductivity and light transmittance, and the process is simple and easy to form a film. Pal et al. [46] used PEDOT:PSS functional inks to form high-resolution conductive micropatterns on SiO_2_ substrates by photolithography, which can be used for high-sensitivity flexible biosensor devices (Figure 3i). On this basis, Pal et al. [47] prepared an SF-based biocompatible and degradable thin-film micro-supercapacitor (µSC) that can withstand 450 bending cycles using the conducting polymer PEDOT:PSS and reduced graphene oxide-doped protein carriers as a biocomposite ink (Figure 3j). These SF-based all-polymer organic devices can be designed as tunable transient devices, providing viable alternatives for flexible and implantable bioelectronics.

Norioka et al. [73] showed that polyacrylamide (PAAm) hydrogels do not fracture when strains of more than 90% or elongations of more than seven times are applied, and they return to their original shape after stress relief. This property gives hydrogels the potential for a wide range of applications. By combining ferritin with functional polymers, researchers are able to fully utilize the charge storage capacity and redox properties of ferritin while imparting additional mechanical strength and electrical properties to the composites. Inspired by the structure and composition of natural ferritin, Wang et al. [48] designed a PAAm-ferritin hybrid hydrogel. PAAm was utilized to provide a chemical cross-linking network, which was applied to wearable flexible strain sensors. Ferritin was uniformly distributed in the crosslinked network and acted as a nano-cage spring, which enhanced the tensile strength of the hydrogel, with a fracture stress of 99 kPa, demonstrating the feasibility of utilizing ferritin to synthesize functional materials (Figure 3k).

Poly(acrylamine hydrochloride) (PAH) is a water-soluble polymer with high hydrophilicity, good chemical stability, and excellent biocompatibility. PAH exhibits cationicity in aqueous solution and can form complexes with a variety of anionic substances such as DNA, proteins, or anionic surfactants. Kim et al. [49] deposited PAH/ferritin nanoparticles (NPs) on SiO_2_-coated wafer substrates to prepare organic field-effect transistors (OFETs). Compared to the metal NP layer used in conventional OFET memories, protein NP-based OFET memories have good programmable storage characteristics, a large storage window ΔVth (>20 V), fast switching speed (10 μs), a low operating voltage (<10 V), a high ON/OFF current ratio (>10^4^), and excellent electrical reliability. The excellent performance after 500 bending cycles proves that ferrites can be effectively used in bendable OFET memory devices as well as silicon-based devices. Also using the electrostatic layer-by-layer assembly method, Ko et al. [50] deposited multilayers composed of anionic ferritin and cationic PAH on Pt-coated Si substrates to obtain PAH/ferritin multilayers and deposited Ag top electrodes on nanocomposite membranes to form nonvolatile memory devices (Figure 3l). The electrostatic layer-by-layer assembly technique significantly increases the charge trap density in the vertical dimension. This significantly improves the storage performance of non-volatile memory devices, which can be generalized to the fabrication of high-performance bioelectronic devices.

Protein–polymer composites combine the biocompatibility and degradability of proteins with the excellent mechanical and electrical properties of polymers. Through innovative composite structure design, highly tunable functional properties of composites can be realized to meet different needs. However, processes such as electrostatic layer-by-layer assembly, while allowing precise tuning of material properties, also increase process complexity and fabrication costs. In addition, the performance of protein matrix composites can be degraded in complex environments, especially in the face of external environmental changes (e.g., humidity and temperature fluctuations), affecting the long-term stability of the devices.

## 3. Applications of Protein-Based Flexible Devices

Protein-based flexible devices are emerging as innovative biomaterials with applications across multiple fields. In sensors, these devices leverage biocompatibility and mechanical properties for accurate physiological signal detection, such as in wearable health monitors. In energy storage, the functional groups of proteins are easy to modify, making them ideal for flexible batteries and supercapacitors. Additionally, protein-based devices show promise in bio-memory for integrating biological and electronic information and in power generation by converting mechanical or environmental energy into electricity, supporting green energy and self-powered systems. Their development is driving innovation in biomaterials and flexible electronics, with potential impacts on healthcare, environmental sustainability, and bioinformation.

### 3.1. Pressure Sensors

Sensors can sense the measured information and can transform the sensed information into electrical signals or other required forms of information output according to certain laws to meet the requirements of information transmission, processing, storage, display, recording, and control [74]. With the rapid development of wearable devices, protein-based flexible sensors are favored for their high sensitivity, flexibility, and good biocompatibility.

#### 3.1.1. Piezoelectric Sensors

Piezoelectric sensors are self-generating electromechanical sensors based on the piezoelectric effect. Its sensitive element is made of piezoelectric material. When piezoelectric material is subjected to force, the internal electrode will produce the phenomenon of polarization and, at the same time, produce opposites charges on two surfaces; when the external force is withdrawn, the material will return to the uncharged state. Compared with traditional piezoelectric materials, protein piezoelectric materials not only have natural flexibility and mechanical strength but also have good biocompatibility. In 1941, Martin first found the polarization phenomenon in asymmetric biological tissues. In recent years, bio-piezoelectric materials have received more and more attention. Many scholars have investigated the microstructure and phase structure of biopiezoelectric materials and have prepared biocompatible high-performance sensors by designing molecular structures, preparing heterostructures and introducing dopants to enhance their physical and chemical properties.

Piezoelectric effects have been widely observed in biological systems. Harvey et al. first reported the piezoelectric properties of silk but did not quantify them [75]. Fukada et al. conducted the first quantitative measurements on the intrinsic shear piezoelectricity of silk fiber bundles [76]. The structural origin of silk piezoelectricity is explained in detail in the literature [77]. The piezoelectric properties of silk have significant scope for applications in miniaturization, wearable sensors, transducers, etc. Joseph et al. [78] designed a piezoelectric transducer based on a silk film using the inherent piezoelectric properties of silk. The main components of the protein structure of SF are alanine (approximately 34%), glycine (approximately 43%), and serine (approximately 14%). Among them, glycine and alanine can exhibit piezoelectric properties. The piezoelectric coefficients of these two amino acids are comparable to or even higher than those of quartz crystals.

Protein piezoelectric materials also offer new solutions for sustainable, self-powered, and high-performance flexible electronics. Protein materials, such as collagen and eggshell membrane proteins, are ideal for the development of piezoelectric sensors and energy harvesters due to their natural biocompatibility, degradability, and excellent piezoelectric properties. These materials are capable of generating an electrical potential difference through mechanical deformation and converting mechanical energy into electrical energy, thus realizing sensing and self-powered functions. In wearable and implantable devices, protein piezoelectric materials not only enhance the sensitivity and environmental friendliness of the devices but also satisfy the requirements for flexibility, lightweight design, and processability, demonstrating broad application prospects in the fields of medical monitoring, energy harvesting, and environmental detection (Table 3).

**Figure 4:**
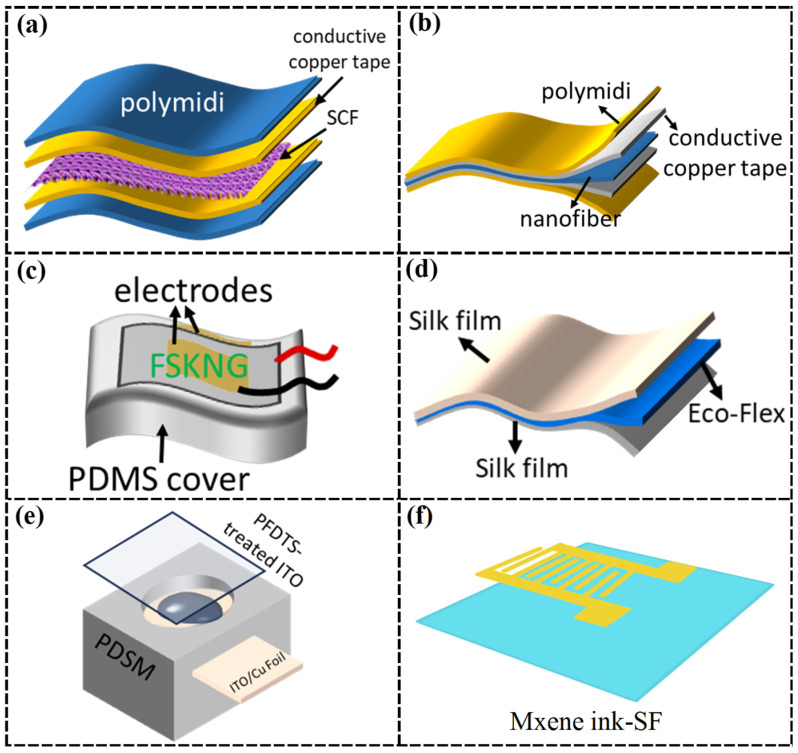
Pressure sensors. (**a**) A flexible and wearable fabric-based piezoelectric nanogenerator [79]. (**b**) A flexible and biomolecular piezoelectric device [80]. (**c**) A human-interactive self-powered wearable biopiezoelectric pressure sensor [81]. (**d**) A stretchable and biodegradable strain and pressure sensor based on Ag NFs/SF [82]. (**e**) A proof-of-concept six-pixel tactile sensor [83]. (**f**) A wearable, breathable, degradable, and highly sensitive MXene/protein nanocomposites-based pressure sensor [84].

Liang et al. [79] utilized soluble eggshell membrane protein (SEP) and polyethylene oxide (PEO) to make biodegradable sensors (Figure 4a). Eggshell membranes, rich in collagen fibers, have piezoelectric properties, giving the sensor a piezoelectric coefficient of 30.9 pm/V and a piezoelectric output of 0.5 V. However, relying solely on the piezoelectric effect of the eggshell membrane limits the output voltage, restricting energy harvesting and signal detection capabilities. To address this issue, Liang et al. [80] enhanced the piezoelectric properties by incorporating SEP/cotton fabric (SCF) (Figure 4b), which forms hydrogen bonds with SEP, improving structural stability and piezoelectric performance. Moreover, a sustainable flexible SEP/SCF piezoelectric sensor was prepared with a maximum output voltage of 1.3 V, a 2.6-fold increase, and enhanced mechanical properties due to an effective self-powered network created by hydrogen bonding. These sensors exhibit excellent sensing performance and environmental friendliness, making them promising for implantable and wearable electronic devices. In a different approach, Ghosh et al. [81] utilized collagen extracted from deep-sea fish skin, which possesses unique properties due to the low-temperature environment, to develop a self-powered wearable bio-piezoelectric pressure sensor (Figure 4c). The self-assembled collagen nanofibers from fish skin exhibit a stable crystal structure and produce a nonlinear electrostrictive effect without polarization treatment. Under 1.8 MPa of external pressure, the sensor generates an open-circuit voltage of 2 V, a short-circuit current of 20 nA, and a power output of 0.75 mWm^−2^, demonstrating its potential as an alternative power source.

Due to the natural flexibility (breaking stress up to 500 kPa, more than five times higher than pure PAM hydrogel), biocompatibility, and degradability of protein piezoelectric materials, as well as the improved charge transfer efficiency that enhances the piezoelectric properties (generating an open-circuit voltage of 2 V). However, the piezoelectric response of proteins is weak compared to piezoelectric ceramics and polymers due to random polarization and lack of ferroelectricity. There has been no ideal solution for realizing controlled large-scale preparation of protein piezoelectric materials.

#### 3.1.2. Capacitive Sensors

Capacitive pressure sensors utilize capacitance-sensitive elements to convert the measured pressure into an electrical output with a certain relationship. When the film senses the pressure and deformation, the capacitance formed between the film and the fixed electrode changes; by measuring the output voltage, creating a certain relationship between the pressure and the electrical signals. It has the advantages of a simple structure, fast dynamic response, good temperature stability, ease of realizing non-contact measurement, etc. It can measure displacement, pressure, thickness, acceleration, rotational speed, liquid level, component content, and other parameters. Proteins and their derivatives are ideal precursors for capacitive sensors because of their easy availability, wide source, excellent mechanical properties, low cost, and environmental friendliness (Table 4).

The development of multifunctional composites suitable for flexible sensors by designing and modulating the structure and properties of the materials can not only enhance the sensitivity, stability, and durability of the sensors but also expand their range of applications [88]. The optimization of the molecular structure, mechanical properties, and electrical conductivity of the materials lays the foundation for the application of sensors in real-time monitoring, wearable devices, and smart systems. At the same time, the combination of new bio-based materials and traditional functional materials to form a high-performance, environmentally friendly, and easy-to-process material system provides an innovative direction for the further development of future sensor technology.

Dong et al. [85] developed flexible capacitive pressure sensors using regenerated silk filament/carbon nanotube (RSF/CNT) conductive films, in which the RSF/CNT film served as the conductive layer and Ecoflex served as the dielectric layer. Under external pressure, the distance between the electrodes decreased, increasing capacitance and enabling pressure-to-capacitance conversion, which makes the sensor suitable for self-powered multifunctional human motion monitoring. However, due to the weak tensile strength of CNTs and the difficulty in translating the properties of individual CNTs into an overall structure, performance degrades in large-scale motion detection [89], which highlights the need for mechanical strength optimization. To address these limitations, Hou et al. [82] replaced CNTs with silver nanofibers (Ag NFs) to create a highly tensile, transparent, conductive, and pliable Ag NFs/SF electrode integrated into a capacitive sensor with high sensitivity (0.01887 kPa^−1^) and a broad monitoring range (35 Pa–700 kPa) (Figure 4d). To further improve performance, Zheng et al. [86] developed a high-performance flexible capacitive pressure sensor using a bio-based protein hydrogel. They synthesized a dual-network (DN) hydrogel from natural ovalbumin (OVA) and polyacrylamide (PAM). The unique DN structure and hydrogen bonding interactions between OVA and PAM are enhanced by Fe^3^⁺ cross-linking, resulting in a significant increase in the breaking stress of the hydrogel, up to 500 kPa, which improves the overall performance of the sensor.

The structural design of sensors is an important strategy for improving the functionality of flexible electronic devices. The mechanical properties, sensitivity, and stability of sensors can be significantly optimized by introducing microsphere structures, dual network structures or unique interface designs. These structural designs can effectively modulate the response properties of the materials to achieve efficient signaling and conversion under a variety of conditions, such as pressure, touch, and light stimulation. Especially in the field of flexible sensors, rational structural design not only enhances the functionality and durability of the device but also expands its potential use in complex application environments, which provides an important direction for the development of high-performance and multi-functional flexible electronic devices.

Wang et al. [87] developed a capacitive sensor with a microstructure formed by microsphere accumulation, using a BSA hydrogel as the dielectric layer and achieving a strain sensitivity of 360.91. The unique microsphere structure of the dielectric layer provides the sensor with high sensitivity, a long lifetime, and good stability. Although interactions between structural proteins maintain high network elasticity and improve the toughness of hydrogels, most protein-based hydrogels, especially those using globular proteins, still exhibit suboptimal mechanical strength and weak responsiveness [90]. To address these limitations, enhancing the mechanical properties of hydrogels remains a critical research focus. An effective approach is to use a DN structure, as demonstrated by Zheng et al. [86], who employed a DN structure with hydrogen bonding and Fe^3^⁺ cross-linking to significantly improve the mechanical properties of hydrogels. Under external pressure, the first brittle network dissipates energy through internal fracture, while the second ductile network maintains structural integrity due to its toughness. The synergistic effect between the brittle and ductile networks increases the strength and toughness of the hydrogel, resulting in a fracture stress of 500 kPa, a sensitivity of up to 2.9 kPa^−1^, and a response time of as short as 18 milliseconds, thus ensuring effective monitoring of physiological signals.

In addition to the single sensing function, the research conducted by Ravi et al. [83] further innovated on the basis of bio-based materials and capacitive sensors by applying bio-based materials to more complex bioelectrochemical liquid bridge sensors and combining them with computer vision and gesture sensing techniques. Innovative touch-to-audio conversion human–computer interfaces were constructed for visually impaired people (Figure 4e). Utilizing the concepts of electric double-layer capacitance and biophotocapacitance, they constructed a six-pixel biophotocapacitive tactile sensor based on the design of microfluidic capacitors that can be matched to six dots in basic Braille characters. Touch-induced asymmetric compression of the liquid bridge between the hydrophobic and hydrophilic electrodes alters the capacitance at the two interfaces, resulting in different touch responses, so this device can be miniaturized to become an ‘energy-autonomous on-skin e-braille reader’ for the visually impaired.

The application of proteins and their derivatives in capacitive pressure sensors demonstrates the advantages of excellent mechanical properties, fast response, versatility and biocompatibility. Through material preparation, structural design, and functional optimization, their capacitance density can be increased by six orders of magnitude compared with ordinary capacitive sensors, and their sensitivity can be greatly improved (2.9 kPa^−1^). However, in long-term use, proteins are susceptible to environmental factors such as temperature, humidity, pH, etc., and may undergo denaturation or degradation, making it difficult to meet the requirements for sensor durability in industrial or medical fields.

#### 3.1.3. Piezoresistive Sensors

Piezoresistive pressure sensors utilize the piezoresistive effect of materials. When the pressure changes, the material produces strain, causing the strain resistance directly diffused on it to produce a change proportional to the measured pressure, which can then be obtained by the measurement circuit as the corresponding voltage output signal. As an important branch of pressure sensors, flexible piezoresistive pressure sensors are characterized by a simple structure, high sensitivity, large working range, fast response speed, and high stability, has well as having potential development needs in the fields of human movement behavior detection, health monitoring, bionic electronic skin development, and human–computer interaction. The natural accessibility, physicochemical properties, and easy processing of proteins, combined with gelatin, Ag NWs, CNTs, and other materials, can significantly improve the mechanical properties of the sensor. They are suitable for use as green and environmentally friendly materials for new-generation bioelectronic devices. The piezoresistive sensors and their performance are shown in Table 5.

High sensitivity and a wide pressure range are key objectives in the design of modern flexible sensors [97]. By introducing innovative materials and optimized structures, superior sensor performance can be achieved across a wide range of application scenarios. Such sensors typically employ functionalized bioproteins, nanomaterials, and composites to form conductive networks with unique microstructures and mechanical properties that enhance their responsiveness and detection range. High sensitivity enables the sensors to capture small pressure changes, while a wide pressure range ensures stable performance under diverse loading conditions, providing important technical support for a wide range of flexible electronic devices.

Silk pectin proteins (SPPs) are important materials for achieving mechanically deformable, biocompatible, and biodegradable devices. Pal et al. [46] prepared high-resolution conductive micropatterns on SPP substrates with PEDOT:PSS conductive ink, which can be used in the preparation of highly sensitive biomolecular sensors. SPP-PEDOT:PSS devices with high charge storage capacity, a wide electrochemical window, and stability under cyclic mechanical deformation are versatile materials for various bioelectronics applications. However, due to the micellar nature of the solution, the film formed by PEDOT-S has a granular structure, and the conductive particles are surrounded by PSS. The presence of this partially insulating layer deviates from the intrinsic properties [98], and the high concentration of acidic groups on the PSS can corrode the electrodes [99] and cause device degradation, which can have a significant impact on device performance. In addition, the ontological toxicity of PEDOT-PSS is usually low, but the preparation process and residues may pose potential health and environmental risks.

Some protein assemblies inherently have a piezoresistive effect; for example, Ha et al. [91] further promoted the combination of nanomaterials and proteins by combining multi-walled carbon nanotubes (MWCNTs) with aerogels made of α-synuclein elongated amyloid protofibrils (eAFs) to develop a porous 3D interconnected structured pressure sensor with high robustness and biocompatibility, with a strain coefficient of 317 and a higher sensitivity than that of metal-, silica-based, and silicon carbide materials. EAFs provide a stable porous 3D interconnected network with high sensitivity to pressure changes, while MWCNTs serve as a reinforcement material to improve the mechanical and electrical properties of the sensor. Embedding MWCNTs into the eAFs aerogel gives the sensor stable piezoresistive sensing performance.

Sensors with excellent mechanical properties are of great significance in the field of flexible electronics, as they are able to maintain excellent structural stability and mechanical strength when subjected to external pressures, while realizing high sensitivity and precise signal response. By combining biological proteins with conductive nanomaterials, these sensors exhibit excellent mechanical toughness, tensile properties, and electrical conductivity, providing reliable technical support for pressure sensing and physiological signal detection in complex environments. The combination of high durability and flexibility makes them promising for a wide range of applications in wearable devices, smart medicine, and industrial devices.

Ling et al. [92] developed graphene/SF nanocomposites using a stable graphene/SF suspension system, achieving ultra-high toughness (611 ± 85% failure strain), high strength (339 MPa), and stiffness (7.4 GPa), making them suitable for piezoresistive pressure sensors. However, due to SF chain relaxation, graphene/SF/Ca^2^⁺ nanocomposites cannot fully restore their resistance after large deformations and thus are preferred as disposable strain sensors for monitoring large deformations with a shorter lifetime (>10 cycles). This limits the application in monitoring a wide range of actions. Further, Wang et al. [93] created transparent carbonized silk nanofiber membranes (CsilkNM), using them as active materials in skin-like pressure sensors with PDMS substrates. CsilkNM-based sensors exhibited superior performance, including a 1000-fold increase in service life (>10,000 cycles) compared to graphene/SF/Ca^2^⁺ composites, along with flexibility, transparency, and suitability for large-scale production. Correia et al. [94] explored CNT/silk-elastin-like protein (SELP) composites, which improved thermal stability and mechanical properties, increasing tensile strength by 1.1 times (101 ± 11 GPa) and strain rate by 6 times (43 ± 17%) compared to pure SELP films. However, the dispersion and homogeneity of CNTs need to be further optimized to prevent the failure of the conductive network at high strains. Reizabal et al. [95] obtained SF nanocomposite films with a uniform distribution of CNTs by ensuring complete CNT dispersion through an ultrasonic bath and magnetic stirring, achieving a sensitivity of 4 MPa^−1^ by forming localized microcapacitors and improving dielectric properties. SF has an inherent power generation capability due to ion- and water-mediated proton hopping transfer [100,101]. Reizabal et al. [96] built on this by using Ag NWs instead of CNTs as a conductive filler to further enhance the piezoresistive response of the material.

Wearable sensors have attracted much attention for their potential applications in smart medicine, human health monitoring, smart sensing, and human–computer interaction systems [102,103], providing technical support for real-time monitoring of vital signs and motion status. These sensors are usually based on flexible materials combined with nanocomposite technology to achieve high sensitivity, excellent mechanical properties, and good biocompatibility. They are capable of adapting to complex body surface deformations and exhibit stable signal output when detecting subtle physiological changes and large movements.

Ke et al. [45] developed a flexible, breathable, and degradable collagen-based skin device using cowhide as a substrate, with in situ growth of polypyrrole (PPy) to impart electrical conductivity and sensing functionality. This device mimics the hierarchical fiber structure of human skin, enabling cross-scale deformation from the nanoscale to the macroscale, which enhances mechanical robustness and sensing performance. However, the mechanical and processing properties of PPy are poor, and the high charge recombination rate due to the small band gap and poor dispersion affects the responsiveness of the sensor [104]. In contrast, MXene materials have gained attention for their tunable electrical, magnetic, optical, thermal, and mechanical properties [105], emerging as a significant advancement in 2D nanomaterials after graphene. Despite their potential, MXene nanosheets suffer from weak interlayer van der Waals forces and hydrogen bonding, leading to poor mechanical strength and durability, as the layers are prone to peeling [106]. To address these issues, Chao et al. [84] developed a biodegradable sericin nanofiber (MXene-SF) membrane combined with MXene ink electrode patterns printed on SF nanofiber membranes, creating a breathable, wearable, and degradable pressure sensor with a wide sensing range (up to 39.3 kPa), high sensitivity (298.4 kPa^−1^ for 1.4–15.7 kPa; 171.9 kPa^−1^ for 15.7–39.3 kPa), and stability over 10,000 cycles (Figure 4f). The porous nanofiber network structure of the MXene-SF membrane and MXene ink-SF electrodes provides a large specific surface area, sufficient roughness, and elasticity, enabling sensitive resistance changes under external pressure.

The rational design of the protein material microstructure can further improve the sensing range (39.3 kPa), sensitivity (4 Mpa^−1^) and stability of the material. By contrast, the piezoresistive pressure sensor prepared with PDMS had a sensing range of only 0–2.6 kPa and a sensitivity of 25.1 kPa^−1^. However, the performance issues of how to simultaneously realize the high sensitivity and large compression range of flexible pressure sensors still need to be solved.

### 3.2. Humidity Sensors

A humidity sensor is a device used to measure the relative humidity in the air and is widely used in many fields such as meteorology, construction, agriculture, medicine, and food processing. Sensors made by combining proteins with moisture-sensitive materials, when water molecules are adsorbed onto the surface of the sensor material, the conductivity, capacitance or voltage of the material will change, thus realizing the detection of humidity.

**Figure 5:**
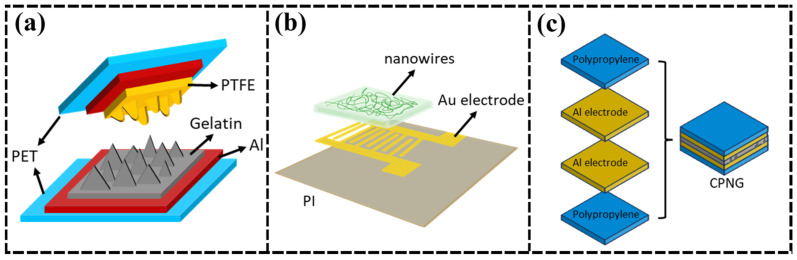
Humidity sensor. (**a**) A self-powered humidity sensor [107]. (**b**) A novel type of electronic sensor made from sustainably produced e-PNs [19]. (**c**) A cost-effective self-powered, eco-friendly, and bio-compatible piezoelectric biopolymer [108].

Liu et al. [107] developed a humidity sensor using electrically conductive protein nanowire (e-PN) films extracted from Geobacter sulfurreducens as the sensing element (Figure 5a). The sensor demonstrated high sensitivity, with a 6% change in relative conductance for every 1% change in relative humidity (RH), making it suitable for real-time monitoring of physiological conditions, such as respiration and skin hydration. However, the weak conductivity of natural proteins limits the current output, resulting in a low self-powered capability. To address this, Vivekananthan et al. [19] developed a SP-PB-HS (Figure 5b). This sensor can generate a V_OC_ of 45 V and an I_sc_ of 250 nA at 5 N. The collagen film enabled humidity sensing through proton conduction and water molecule adsorption, which can be used for high-sensitivity humidity monitoring; however, its V_OC_ response decreased from 45 V to 4 V with increasing RH, thus limiting voltage output. To overcome the issue of reduced triboelectric nanogenerator (TENG) output in high RH environments, Chang et al. [108] constructed biocompatible TENGs by using gelatin/glycerol and polytetrafluoroethylene (PTFE) as friction electric layers (Figure 5c). At high RH, the hydration of gelatin caused the formation of protonated amino groups, which attracted hydroxide ions on the side chain, increasing surface charge density and making gelatin-based TENGs self-powered humidity sensors with good stability and durability. These devices show promising applications in areas such as smart environmental monitoring and green energy harvesting (Table 6).

Vivekananthan’s research demonstrated that cotton fabrics have significantly lower moisture absorption than collagen. The polar amino acid sequence of collagen enhanced the humidity response through chemisorption and physisorption, which significantly enhanced the sensitivity of the humidity sensor. However, the current output still suffers from gradual decay in humid environments. For example, the sensor developed by Vivekananthan et al. showed a clear trend of decreasing output current during prolonged operation under constant humidity conditions. In contrast, the sensor proposed by Liu et al. showed excellent stability under the same conditions, and its output current showed almost no decay. This difference suggests that the long-term stability of protein-based humidity sensors still needs to be further optimized to overcome the current decay problem.

### 3.3. Energy Storage Devices

Energy storage components are a class of devices capable of storing electrical energy and releasing it when needed. The development of high-performance, low-cost, flexible electronic devices is a key prerequisite for emerging applications in energy storage, conversion, and sensing systems. Proteins, as important biomaterials with high biological, chemical, and physical activities, but with simpler structures than high-dimensional biological tissues, can be used as components of different energy storage devices, such as electrolytes, separators/intermediate layers, catalysts, and adhesives [109]. It has become a hot spot for bioelectronics research in recent years.

#### 3.3.1. Supercapacitor

As a high-performance energy storage device, supercapacitors have attracted much attention in the modern energy field due to their excellent energy density, fast charging and discharging capabilities, and long-term cycling stability. Supercapacitors based on functionalized materials show great potential for performance optimization, especially in terms of improved electrical conductivity, specific surface area, and electrochemical activity, which provide important support for the development of efficient energy storage and wearable devices. Through the introduction of protein material design and innovative manufacturing processes, such devices possess multiple advantages of environmental friendliness, degradability, and efficient energy storage, and have a wide range of applications in the fields of green energy and smart devices (Table 7).

**Figure 6:**
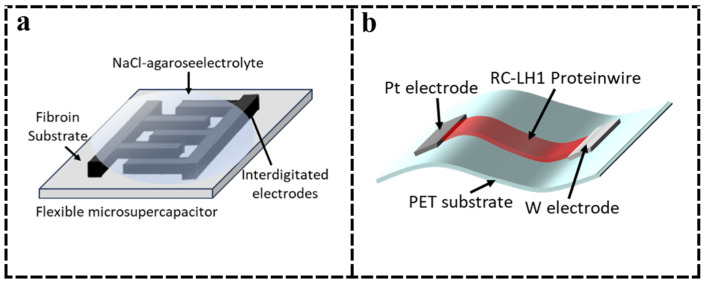
Energy storage device. (**a**) A green and sustainable flexible microsupercapacitor based on a conducting polymer [47]. (**b**) An all-printed, solid state, flexible photoelectro protein micro-capacitor [111].

Pal et al. [47] demonstrated a fully organic and degradable SF-based biocompatible µSC based on the conductive polymer PEDOT:PSS and graphene-doped SF carrier as a photopatternable biocomposite ink (Figure 6a). Capacitance values up to 148.3 F/g can be achieved with reversible capacitance characteristics and comparable power densities to conventional devices. However, the chain structure of PEDOT:PSS is an intertwined disorder in suspension, which can lead to low controllability during chemical reactions, thus reducing its electrochemical performance [112]. Therefore, PEDOT:PSS suffers from low conductivity compared to other hole-transporting materials, which seriously affects the performance of the sensor. Song et al. [110] manufactured a regenerated SF-based PWSC electrode, PPy/RSF/MWCNTs-2, to replace PEDOT:PSS. The weight-specific capacitance could reach 547.5 F/g. The interconnected conductive network inside the MWCNTs and the relatively open structure of the external PPy can accelerate the penetration of the electrolyte, which can help to realize the redox reaction adequately and make the PPy/RSF/MWCNTs-2 exhibit excellent electrochemical performance. However, the composite and mechanism of action of PPy with other materials are not completely clear, and there are serious structural degradation and counter-ion effects during charging and discharging [113], which limit its application in high-performance supercapacitors. Protein-based supercapacitors, with their excellent performance, can be widely used in industrial equipment, high-efficiency energy storage devices, and aerospace applications.

The average size of the weight-specific capacitance of supercapacitors prepared from inorganic materials (e.g., MXene materials, manganese dioxide, activated carbon materials) is roughly in the range of 100–400 F/g. In contrast, the high specific surface area, porous structure, and large number of functional groups in the molecules of protein materials provide more active sites for charge storage. It makes its weight-specific capacitance up to 547.5 F/g and excels in environmental friendliness and biocompatibility. However, its structural degradation during charging and discharging limits performance stability. Although the permeability and redox reaction efficiency of the electrolyte were optimized by multi-material composites, the unclear material composite mechanism and the complexity of the preparation process are still the main challenges.

#### 3.3.2. Optical/Bio-Hybrid Devices

Photovoltaic/biohybrid devices combine the unique properties of photovoltaic conversion technology and biomaterials, showing great potential for energy storage and photovoltaic applications. By integrating light-trapping systems and protein materials, such devices realize efficient light energy conversion and storage functions while being environmentally friendly and sustainable, providing innovative ways to develop new energy devices and photovoltaic technologies.

Paul et al. [111] used electrohydrodynamic printing technology to print RC-LH1 protein inks on PET film substrates to obtain bio-hybridized optoelectronic protein microcapacitors with high performance and stability (Figure 6b). Due to the bacterial chlorophyll and carotenoid pigmentation of the RC-LH1 complexes, the inks have light-trapping capabilities in the near-ultraviolet, the blue and green regions of the visible spectrum, and in the near-infrared, providing unique light conversion and storage capabilities. The optimized procedure enables a specific capacitance of 110 mF/g and a scan rate of 10 mV/s.

Protein-based photovoltaic/biohybrid energy storage devices achieve high specific capacitance and excellent mechanical stability with photoresponsive capability under low light. However, the device performance is limited by the intrinsic properties of the protein material, such as the photogenerated charge being limited by a slow diffusion process, resulting in an actual photosynthetic efficiency of only 3–6%.

### 3.4. Electronic Bio-Memory Devices

After millions of years of evolution, natural materials possess near-perfect structure and function, with electron transfer being one of the most fundamental and complex biological processes [114]. Proteins consist of at least one polypeptide chain with a specific three-dimensional structure. In particular, some redox proteins, such as ferritin, myoglobin, and lysozyme, typically contain multiple charge trapping sites in their three-dimensional structures and play key roles in a range of bioelectrochemical processes. The reversible redox behavior, electrically insulating properties, and resistive switching effects of proteins and peptides make them promising memory materials and are expected to be ideal candidates for memristors and transistor memory devices.

#### 3.4.1. Memristor

As the limits of transistor technology are approached, the feature sizes of integrated circuit transistors have been reduced to very close to the smallest physically achievable channel lengths, and it is becoming increasingly difficult to meet the expectations outlined by Moore’s Law. In 2008, HP Labs realized the first memristor in a TiO_2_ material [115], which laid the groundwork for subsequent research into the structure and circuitry of memristor devices [116]. As a new type of electronic component that enables high-density, multi-functional, low-power, and multi-level data storage, the memristor has many excellent properties that can be used to develop new neural and non-von Neumann computing systems, which are expected to revolutionize information processing technology [117]. Among the wide range of memristors, protein-based memristors are regarded as ideal devices for building next-generation high-performance electronic products due to their controlled degradability, excellent performance, abundant raw materials, low cost, and biocompatibility (Table 8).

**Figure 7:**
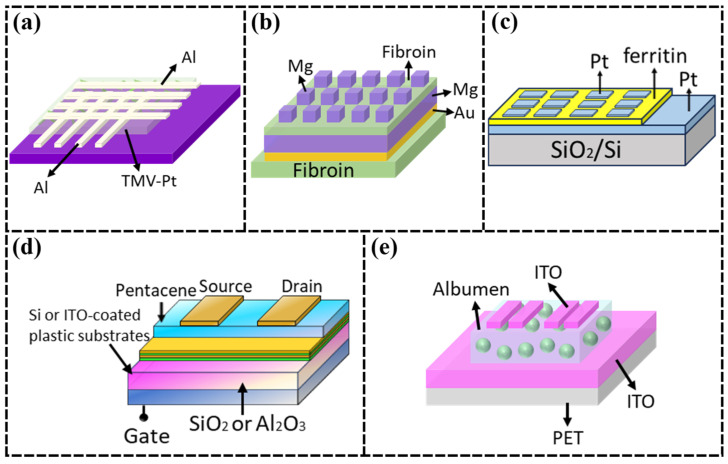
Electronic bio-memory device. (**a**) A new memory effect function in the hybrid system composed of TMV conjugated with Pt [118]. (**b**) Physically transient resistive switching memory nanodevices based on silk protein [44]. (**c**) A biocompatible memristor device based on natural ferritin has been fabricated [37]. (**d**) A novel type of OFET memory device based on ferritin NP multilayers [49]. (**e**) Flexible ITO neuromorphic transistors, gated [121].

A non-volatile memristor is a resistive switching device with a memory function that is capable of maintaining a stable HRS or LRS under power-on or power-off conditions, demonstrating excellent data storage capability and reliability. Yang’s team developed a non-volatile electronic bio-memory device using tobacco mosaic virus (TMV) coupled with Pt [118] (Figure 7a). The TMV-Pt composite exhibits electrical bistability, transitioning between conductive and non-conductive states under voltage control. At higher voltages, charge tunneling replaces thermal ion injection as the conduction mechanism, offering a novel approach to fabricating functional electronic devices with biomaterials. However, reliance on charge tunneling may lead to high energy consumption. To address this, Moudgil et al. [119] created a non-volatile flexible resistive switching memory device using S-layer proteins (Slp), which employ a charge trapping and releasing mechanism to avoid energy-intensive charge tunneling. This device (Al/Slp/ITO/PET) demonstrates stable bistable memory behavior, switching between low-resistance (LRS) and high-resistance (HRS) states. The resistive switching mechanism involves trapping and releasing charges in the Slp layer, forming conductive pathways under voltage. However, the stability of these pathways is limited by the concentration of trapped charges.

SF is easily bonded to metallic materials. In addition, the amino acid chains in the SF structure have good charge trapping and releasing abilities, which can achieve non-volatile memory behavior through redox reactions as well as the formation and breakage of conductive filaments. Hota et al. [120] used SF for non-volatile transparent bio-memory thyristors. The redox properties of SF were utilized to form or disconnect conducting filaments. This approach achieved reliable resistive switching, laying the groundwork for further research. Wang et al. [43] developed an Ag/SF/Au-based resistive-switching memory with a high switching ratio (10⁵) and retention time (>10^4^ s), but the device failed to meet solubility requirements. To overcome this, Wang et al. [44] used Mg electrodes to achieve solubility and biocompatibility while maintaining an OFF/ON ratio > 10^2^ and retention time > 10^4^ s (Figure 7b). These SF-based devices, dissolvable in water or buffer solutions, are promising for transient electronics, secure data storage, and biocompatible applications. Gogurla et al. [41] integrated gold nanoparticles (Au NPs) into an SF switching layer to achieve conductive network control through Coulombic interactions and prepared a transparent flexible device with a low operating voltage (±2 V) and a high switching ratio (10^6^). The use of Au NPs improves the electrical performance of the device while maintaining flexibility and transparency, demonstrating the potential of metallic materials in the application of metallic materials in bio-based memory devices.

Mixed-mode memory is an innovative memory device that combines the characteristics of non-volatile and volatile storage, offering diverse functionality and greater flexibility by integrating two storage modes in the same device. Its storage behavior can be dynamically switched according to the adjustment of the applied voltage or current to adapt to different application requirements.

The Pt/ferritin/Pt sandwich structure biocompatible memory device prepared by Zhang et al. [37] exhibits not only non-volatile memory switching behavior but also volatile threshold switching behavior (Figure 7c). The inter-switching of the two behaviors can be achieved by controlling the magnitude of the compliance current (CC). The resistive switching behavior of the device was observed by releasing ferric ions from the ferritin shell under the action of an electric field. The ferric ions build conductive pathways in the ferritin film, which have limited size and strength and are prone to fracture at low CC preset values, leading to the volatile switching behavior of the device. When the CC preset is high enough, a stable conductive pathway can be formed, exhibiting non-volatile memory switching behavior.

Protein-based memristors have excellent memory storage performance, and their relevant parameters meet marketable requirements. However, the switching characteristics of protein-based memristors, especially the device retention characteristics and erasure speed, are still not comparable to those of inorganic storage media. The properties of different types of protein materials vary greatly, and different production specifications of the same type of material also yield different performance, so it is impossible to achieve a balanced performance [114].

#### 3.4.2. Transistors

Non-volatile storage transistors enable long retention and fast readout of data by trapping and releasing charge in the gate dielectric layer. Compared with traditional storage devices, non-volatile storage transistors have advantages such as low power consumption, high switching speed, and excellent electrical stability. In recent years, with the continuous progress of material science, the introduction of nanostructures and bio-based materials has provided a new direction for the performance enhancement of nonvolatile storage transistors. The transistors involved in the construction of protein materials and their properties are shown in Table 9.

Kim et al. [49] have successfully prepared OFET memory devices using highly stable ferritin nanoparticle (NP) multilayers with a pentacene semiconductor material (Figure 7d). OFETs exhibit excellent mechanical bending stability, further demonstrating their potential in the field of flexible electronics. Compared with the metal NP layer used in conventional OFET memory devices, protein NP-based OFET memory devices have good programmable storage characteristics with a large storage window ΔVth (>20 V), fast switching speed (10 μs), low operating voltages (<10 V), high ON/OFF current ratios (>10^4^) and excellent electrical reliability. Due to the reversible Vth displacement induced by charge trapping and release in the ferritin gate dielectric layer, the device shows non-volatile storage characteristics, replacing metal NPs in transistor memory devices.

Neuromorphic transistors are a class of innovative devices capable of mimicking biological synaptic behaviors through the coupled modulation of charges and ions to achieve the simulation of synaptic plasticity. Compared to conventional electronic devices, neuromorphic transistors are able to simulate complex interactions between neurons at the hardware level, demonstrating the potential of brain-like computing [17]. Such devices have significant advantages in terms of low power consumption, high integration, and compatibility with biological systems, providing a novel pathway for building next-generation neuromorphic computing systems, smart medical devices, and brain-like artificial intelligence.

Wu et al. [122] prepared indium-zinc oxide (IZO) synaptic transistors using a natural protein liquid film with high proton conductivity as the electrolytic medium, ITO glass as the substrate and bottom-gate electrode, and IZO as the channel layer, source, and drain. Synaptic plasticity was successfully simulated, demonstrating that protein-gated synaptic transistors are promising for building biocompatible synaptic electronics and brain-inspired systems. However, the final finished device is a rigid structure, and the ITO glass has low mechanical strength and is susceptible to external oxidation and corrosion [123]. On this basis, Li et al. [121] present a protein-gate-controlled flexible ITO ion/electron-coupled neuromorphic transistor prepared on a flexible PET substrate (Figure 7e). The transistor exhibits excellent mechanical stability under bending stress and unaffected synaptic plasticity behavior. The device successfully mimics three types of spike-time-dependent plasticity learning rules and has the ability to mimic neurotransmitter release behavior. In addition, the device realizes algebraic operations (addition, subtraction, multiplication, and division) on a single flexible transistor for the first time, demonstrating the potential applications of neuromorphic devices in areas such as wearable cognitive platforms, smart robots, and neuroprostheses.

Compared to the storage window (1–2 V) of conventional silicon-based memories, protein-based transistors provide deep, stable charge traps due to their redox-active core. The multilayer structure design enhances the charge storage density in the vertical direction. Thus, they have a larger storage window (>20 V), and the performance can be tuned by molecular design. However, the immobilization and functionalization of proteins require sophisticated biotechnology, which significantly increases the manufacturing cost and process difficulty.

### 3.5. Generator

In recent years, power generation devices based on biomaterials have gradually become an important research direction in the field of energy harvesting. Humidity-driven generators and TENGs are two novel energy conversion technologies that utilize moisture in the air or mechanical friction to convert ambient energy into electricity, which can be used to drive low-power devices or stored for subsequent use and are indispensable components of self-powered energy systems, advancing the development of sustainable energy technologies. The role of proteins, as a bio-based material, in enhancing TENG performance, self-healing capabilities, and multifunctionalization further broadens the prospects for the application of these technologies in the fields of wearable electronic devices, micro-energy harvesting, and so on (Table 10).

#### 3.5.1. Humidity Driven Generators

A moisture generator as a new type of power supply, can absorb moisture in the air, under the action of the hydrovolt effect and concentration gradient to realize the ion directional movement and produce direct current output [128]. Previously, scientists have been utilizing water in the air for energy. For example, in 2015, Qu et al. [129] reported the use of graphene oxide membranes to achieve humidity-to-electricity conversion and have since continued to advance the power generation efficiency of this process. Alternatively, the process of water evaporation into the air can be used to generate electricity. In 2017, Jun et al. [130] found that water evaporation on the surface of nanostructured carbon materials is also capable of generating voltage. With the help of inexpensive carbon black lamellar materials, a sustainable voltage of nearly 1 V can be generated at room temperature using water evaporation. Moisture generators capture energy from the environment and can be used as a power source for portable electronics to provide electrical power.

**Figure 8:**
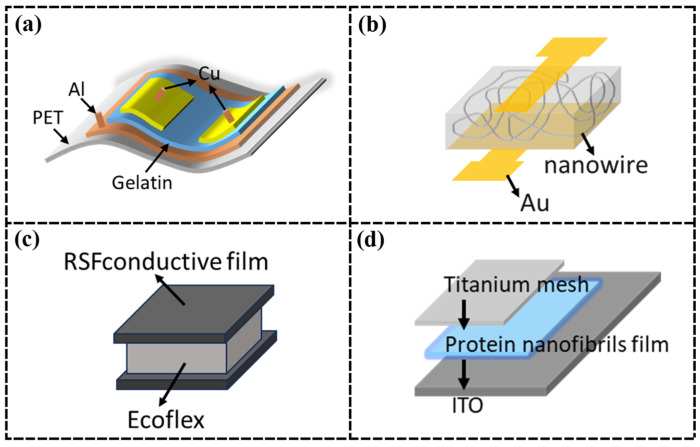
Generators. (**a**) A flexible moisture-induced device [124]. (**b**) An electric generator using a thin film of protein nanowires [125]. (**c**) A flexible capacitive-type pressure sensor assembled from an RSF/CNT film [126]. (**d**) A PN-MEG based on protein nanofibrils derived from milk β-lactoglobulin [85].

Moisture-responsive properties of proteins generate potential differences and drive electron flow [131]. Mandal et al. [124] developed a flexible moisture-induced energy device by sandwiching collagen gelatin as an active layer between two metal contacts with different work functions (Figure 8a). The power generation from these devices in the presence of 90% relative humidity is 5.52 µW/cm^2^. The device generates electricity by absorbing water molecules from a humid environment to produce protons and transferring them to metal contacts by forming conductive paths through hydrogen bonds, which can be used to monitor changes in humidity by measuring the power generated. Liu et al. [125] further expanded the possibilities of moisture-generating materials by making thin-film devices using nanometer-scale protein wires (Figure 8b). The protein wires were harvested from the microbe Geobacter sulfurreducens, which can generate electricity continuously in humid environments. Water molecules in the air ionize when adsorbed on the surface of the nanowires, and the ionized water molecules form a charge on the surface of the nanowires and drive a closed-loop current, which can generate an open-circuit voltage of about 0.53 V on a 7 µm-thick film with a power density of 4 mW cm^−3^ and a stable discharge of more than 20 h. The nanowires can be used for a wide range of applications, including the production of microbial protein nanowires. However, the current extraction process of microbial protein nanowires is complicated, and the yield is not high. Although sustained energy can be obtained by using protein nanowires, the power output of moisture generation is still poor compared to other renewable energy devices.

Therefore, Liu et al. [126] explored a more efficient humidity energy conversion scheme based on the study of a moist-electric generator (PN-MEG) (Figure 8c). Thin films prepared from β-lactoglobulin-derived protein nanoprincipal fibers from milk could generate an open-circuit voltage of up to 0.65 V and a short-circuit current of 2.9 µA, and could produce a maximum power density of 38.88 μW·cm^−2^, achieving the highest power density compared with previously reported biomaterials-based moist-energy generators. Water molecules adsorbed on protein membranes ionize and migrate in response to a humidity gradient, forming an electric field and a closed-loop current, which leads to the continuous power output from the PN-MEG. The β-lactoglobulin nanofibers demonstrated higher power output capability compared to gelatin and nanoscale protein wire materials, proving the great potential of protein-based wet power generation devices for efficient energy harvesting.

Compared with the traditional water-based energy harvesting technology in which only intermittent and brief (<50 s) energy output can be generated, the hydrophilic groups in proteins can effectively adsorb water molecules and ionize ions to form stable ion-conducting channels and maintain high ion-conducting efficiency, thus prolonging the discharge time (>20 h). However, the disadvantages of protein materials, such as lower power generation efficiency and energy density and high cost, limit their widespread application.

#### 3.5.2. Triboelectric Nanogenerator

TENG is a new energy technology that converts mechanical energy into electrical energy through friction electric effect and electrostatic induction, which is lightweight, flexible, efficient, and environmentally friendly [132]. In recent years, natural proteins, as a type of bio-based material with unique physicochemical properties, have been gradually introduced into the design of TENGs. With rich functional groups, excellent flexibility, and biocompatibility, protein materials play an important role in enhancing the output performance of TENGs, strengthening the self-healing ability and realizing multifunctionalization. These properties not only provide new possibilities for the application of TENGs in fields such as micro-energy harvesting, self-powered devices, and wearable electronics but also lay a solid foundation for the development of sustainable energy technologies based on natural materials.

In the study of flexible TENGs, the innovative design of bio-based materials provides a wide scope for performance enhancement and functional diversification. Dong et al. [85] also applied the prepared RSF/CNT conductive films in wearable friction electric nanogenerators (Figure 8d). In addition to capacitive pressure sensors, multifunctional RSF/CNT films can also be designed into SF-based friction electric nanogenerators (SF-TENG) for self-powered sensing and energy harvesting. By coating Ecoflex on RSF/CNT films, single-electrode friction electric nanogenerators have been prepared, and the V_oc_, I_sc_, and Q_sc_ can reach 13.5 V, 26.7 nA, and 4.5 nC, respectively. However, since the durability and mechanical strength of silk-based electronic materials are usually lower than those of conventional electronic materials, there is still a need to focus on their durability and structural stability in the further development and application of silk-based electronic devices.

In order to solve the problem of how the performance of TENG is fatally damaged when the electrode layer is damaged or ruptured when conventional polymer materials are applied to TENGs [133], Han et al. [128] prepared a flexible and self-healing single-electrode TENG by introducing gelatin into polyacrylic acid (PAA). Gelatin has good biocompatibility and degradability [134,135], and the combined effect of its triple-helix cross-linking structure and the reversible cross-linking network formed by dynamic hydrogen bonding between the PAA-gel molecules helps improve the mechanical properties of the hydrogel. When the hydrogel was cut into two halves, dynamic hydrogen bonding between PAA molecules served as a dynamic intersection point. Meanwhile, reversible non-covalent coordination interactions of the PAA-NaCl hybridization network enable the ionic hydrogel to rapidly self-heal at room temperature. Meanwhile, a sensor array simulating electronic skin was prepared using an 8-pixel array, which can make conformal contact with the skin surface, generating an output voltage of 10 V under light touch, and the generated electrical energy can light up a diode. The excellent self-healing properties, good mechanical stability,. and sensing stability of the self-healing friction electric nanogenerator are of great significance for the development of flexible electronics.

Protein-based TENGs have significant advantages in terms of environmental friendliness, biocompatibility, and flexibility (800% elongation) and are suitable for biomedical and wearable device applications. However, inorganic-material TENGs perform better in terms of output performance (e.g., voltage, charge density) and is suitable for high-power-demand scenarios.

## 4. Conclusions

Protein-based flexible devices demonstrate great potential in the field of flexible electronics through their highly functionalized molecular structures and unique chemical activities. Protein materials such as filipin, ferritin and collagen are ideal substrates for flexible devices due to their excellent mechanical properties, biocompatibility, environmental friendliness and tunable molecular assembly properties. At present, protein-based materials have been successfully applied to pressure sensors, humidity sensors, energy storage devices, electronic bio-memory devices and power generation devices in many directions. These materials not only endow flexible electronic devices with excellent mechanical and electrical properties but also promote the development of flexible electronic technology in the direction of environmental protection and functionalization.

In the future, the molecular engineering of protein materials and the structural design of flexible devices will still have extensive avenues for development. In-depth study of the mechanism of action of proteins, the synergistic effects of functionalization modifications and composite materials, and their long-term stability in complex environments will be the focus of future research. With the synergistic progress of materials science, biotechnology, and electronics technology, protein-based flexible devices are expected to open up new directions in the fields of healthcare, green energy, smart wearables, and environmental protection, and to promote the development of flexible electronic devices towards biologization, intelligence, and sustainability [136,137], which, in turn, will provide innovative ideas and solutions for the development and application of flexible electronic devices.

## Figures and Tables

**Table 1:** Electrical properties of proteins themselves and their applications.

Author	Type	Material	Performance	Application Scenario
Silva et al. [18]	Pressure-sensitive properties	Collagen-chitosan	Piezoelectric strain constant is 0.212 pC/N	Coating of cardiovascular prostheses, support for cellular growth, and in systems for controlled drug delivery
Vivekananthan et al. [19]		Collagen-coated cotton fabric	Energy harvesting and humidity sensing	Self-powered humidity sensors
Zeng et al. [20]	Insulation properties	Collagen + Ag/Bio film/ITO or Ti	HRS/LRS switching ratio of 10 (ITO) and 100 (Ti)	Non-volatile memory, rigid devices
Hosseini et al. [21]		Mg/collagen/ITO	Flexibility and durability, completely soluble in water	Defense data storage, classified information analysis
Lin et al. [22]		Keratin	Switching ratio > 10^3^, holding time > 10^4^ s, dissolves within 30 min	Non-volatile memory
Wang et al. [23]		SF, PET	Mobility of 23.2 cm^2^/V·s, operating voltage of −3 V	OTFTs

**Table 2:** Summary of devices in which proteins are complexed with other materials.

Author	Type	Material	Performance	Application Scenario
S. Bera et al. [32]	Carbon and 2D materials	ITO/CNT–CdS-silk composite/Al	High charge storage capacity, adjustable memory window, flexible and transparent	Floating gate memory device
Lei et al. [33]		Mn-doped N-containing carbon materials + collagen	High specific capacitance, long life, resistance to extreme conditions	Electrode material for supercapacitors
Selvam et al. [34]		PPy + tungsten disulfide nanoparticles + collagen	Areal capacitance of 348 mF/cm^2^, biocompatible and highly stable	High-performance SC, biomedical devices
Elmas et al. [35]	Metal ion	Ferritin, Ag, Cu	Higher operation cycles, durability and reusability	Ferritin-based bionanocages
Ichikawa et al. [36]		Ferritin	Large memory window, retention time of over 4000 s	LTPS-TFT flash memory
Zhang et al. [37]		Pt/ferritin/Pt	Fast operation speed and low power consumption for 3D integration	Information storage, logic circuits, neuromorphic computing
Andonegi et al. [38]	Nanomaterials	Collagen/Ag NWs	Electric conductivity of 0.0515 S/cm	Antimicrobial material, resistive sensor
Chen et al. [39]		SF, Ag NWs	Highly conductive, excellent mechanical properties, transparent film, degradable	Transient electronic devices, environmentally friendly wearable devices
Lee et al. [40]		SF, gold nanoparticle	High sensitivity, real-time monitoring	Silk plasma absorption sensor, body fluid monitoring
Gogurla et al. [41]		SF, gold nanoparticle	Flexible transparent resistor switching memory with good memory performance	Transparent flexible memory
Gogurla et al. [42]		Gold-filament composites, ZnO films	Excellent photoelectric performance and high sensitivity	MSM lateral photodetectors
Wang et al. [43]		Au film, SF	Ultra-lightweight design	Lightweight resistive switching memory
Wang et al. [44]		Au/Mg/SF	Transiently dissolvable, excellent resistive switching performance	Transient resistor switching memory
Ke et al. [45]	Polymers	Cowskin	High water vapor permeability, durability, cross-scale testing	Wearable sensory skin for physiological signal monitoring
Pal et al. [46]		PEDOT:PSS functional ink comprised, SF	Conductive micropatterning, flexible, highly sensitive, degradable	Biomolecular sensors, flexible and implantable bioelectronics
Pal et al. [47]		PEDOT:PSS, reduced graphene oxide, SF	Miniature supercapacitors, biocompatible, tunable	Transient electronic devices, flexible electronic devices
Wang et al. [48]		PAAm-Ferritin Hybrid Hydrogel	Biocompatible and highly conductive	Wearable flexible strain sensors
Kim et al. [49]		PAH/ferritin multilayer membrane	Stable performance after 500 bending cycles	Flexible OFET memory devices, silicon-based devices
Ko et al. [50]		PAH/ferritin multilayer membrane	Non-volatile memory, high storage capacity	High-performance bioelectronic memory devices

**Table 3:** Piezoelectric sensors and their performance.

Author	Type	Material	Performance
Harvey et al. [75]	Studies on the origin of the piezoelectric properties of proteins	Silk protein	Silk piezoelectric properties were reported but not quantified
Fukada et al. [76]		Silk protein	Quantitative study of the intrinsic shear piezoelectricity of silk fibers
Joseph et al. [78]		Silk protein	Alanine and glycine in silk exhibit piezoelectric properties
Liang et al. [79]	Electronic devices based on protein piezoelectric materials	SEP	Produces a significant output response to external forces
Liang et al. [80]		SEP/SCF	Self-powered network, excellent electrical properties and environmental friendliness
Ghosh et al. [81]		Fish skin	Can generate 2 V open-circuit voltage and 20 nA short-circuit current with a power density of 0.75 mWm^−2^

**Table 4:** Capacitive sensors and their performance.

Author	Type	Material	Performance
Dong et al. [85]	Material preparation and optimization	RSF/CNT	Self-powered sensors
Hou et al. [82]		Ag NFs/SF laminated film	Highly stretchable, transparent, conductive, wide monitoring range (35 Pa–700 kPa), high sensitivity
Zheng et al. [86]		OVA/PAM DN	High sensitivity (2.9 kPa^−1^), short response time (18 ms), fracture stresses of up to 500 kPa
Wang et al. [87]	Structural design	BSA hydrogel	Microspheres accumulate to form microstructures, providing high sensitivity, long life and good stability
Zheng et al. [86]		OVA/PAM DN	High fracture stress, short response time (18 ms)
Ravi et al. [83]		Photosynthetic bacterium	Liquid bridge design, electrical signal of up to 2 V under light excitation, supports Braille character recognition

**Table 5:** Piezoresistive sensors and their performance.

Author	Type	Material	Performance
Pal et al. [46]	High sensitivity and wide pressure range	SPP, PEDOT:PSS	Wide potential window from −1.0 V to 1.6 V, sensitivity of 21.169 µA/mM.cm^2^
Ha et al. [91]		αS/MWCNT composite aerogel	Accurately recognizes different pressure levels and responds quickly
Ling et al. [92]	Sensors with excellent mechanical properties	Graphene/SF nanocomposites	High toughness, high strength, high stiffness, signal stability
Wang et al. [93]		Carbonized silk nanofiber film	Sensitivity of 34.47 kPa^−1^, Lower detection limit of 0.8 Pa, service life > 10,000 cycles, short reaction time
Correia et al. [94]		SELP/CNTs nanocomposite film	High sensitivity, non-cytotoxic
Reizabal et al. [95]		SF/SNW nanocomposites	Sensitivity of 4 MPa^−1^, high signal stability and durability
Reizabal et al. [96]		SF/CNT nanocomposites	High sensitivity and good signal stability
Ke et al. [45]	Wearable sensor	Collagen/PPy skin device	Sensitivity of 0.144 kPa^−1^, rapid response (200 ms), high cyclic stability (15,000 cycles)
Chao et al. [84]		MXene-SF film, Mxene inks-SF electrodes	Highly sensitive, wearable, breathable, biodegradable

**Table 6:** Humidity sensors and their performance.

Author	Type	Material	Performance
Liu et al. [107]	Adsorption and conductivity change humidity sensors	E-PNs film	Real-time monitoring of physiological conditions such as respiration and skin hydration
Vivekananthan et al. [19]	Electric field and ion transport humidity sensors	Collagen film	Significant change in open-circuit voltage with humidity change
Chang et al. [108]	Friction effect type humidity sensors	Gelatin/Glycerin and PTFE	Self-powered humidity sensor with good stability and durability

**Table 7:** Energy storage devices and their performance.

Author	Type	Material	Performance
Pal et al. [47]	Supercapacitor	PEDOT:PSS + graphene + SF	Capacitance is 148.3 F/g, biodegradable, biocompatible
Song et al. [110]		PPy + RSF + MWCNTs	Area ratio capacitance of up to 8704.7 mF·cm^−2^
Paul et al. [111]	Optical/bio-hybrid devices	RC-LH1 protein + PET	Capacitance is 110 mF/g, light-trapping capability

**Table 8:** Memristors and their performance.

Author	Type	Material	Performance
Yang et al. [118]	Non-volatile memristor	TMV-Pt	Bistable storage, high switching ratio
Moudgil et al. [119]		S-layer protein	Flexible device, bistable storage
Hota et al. [120]		SF	Transparent, non-volatile memristor
Wang et al. [43]		SF	Ultra-lightweight and biocompatible
Wang et al. [44]		SF	Switching ratio > 10^2^, holding time > 10^4^ s, dissolved in deionized water
Gogurla et al. [42]		SF + Au nanoparticles	Low operating voltage (±2 V), high switching ratio
Zhang et al. [37]	Mixed-mode memristor	Ferritin	Mode switching by adjusting the compliance current

**Table 9:** Transistors and their performance.

Author	Type	Material	Performance
Kim et al. [49]	Non-volatile storage transistors	Ferritin nanoparticles	ΔVth > 20 V, switching speed 10 μs, switching ratio > 10^4^
Wu et al. [122]	Neuromorphic transistor	Protein liquid film	Rigid device
Li et al. [121]		Egg white	Mimics neurotransmitter release behavior, excellent mechanical stability under bending stresses

**Table 10:** Generators and their performances.

Author	Type	Material	Performance
Mandal et al. [124]	Humidity-driven generators	Gelatine	Power density of 5.52 μW/cm^2^, can be used to monitor breathing patterns
Liu et al. [125]		Protein nanowire	Open circuit voltage of 0.53 V, power density of 4 mW cm^−3^
Liu et al. [126]		β-lactoglobulin	Power density of 38.88 μW/cm^2^, open-circuit voltage of 0.65 V
Dong et al. [85]	TENG	RSF/CNT film	Pressure sensing and energy harvesting
Han et al. [127]		Gelatine, PAA	Self-healing, 800% elongation, 10 V output voltage
